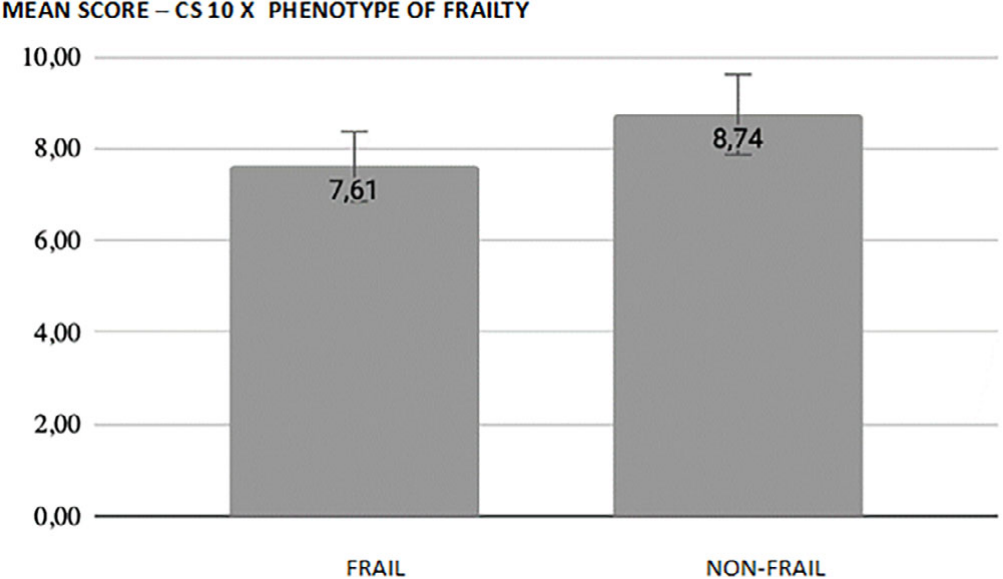# Do patients with early‐stage cognitive impairment have an increased risk for developing frailty syndrome? A cross‐sectional study

**DOI:** 10.1002/alz.089037

**Published:** 2025-01-09

**Authors:** Pedro de Castro Lopes, João Carlos Barbosa Machado, Maira Tonidandel Barbosa, Bianca Pessoa Aguiar, Júlia Caroline Barbosa de Souza, João Pedro Neres Antunes Ferreira, Luana Rodrigues Garcia, Amanda Aparecida Oliveira Leopoldino

**Affiliations:** ^1^ Faculdade Ciências Médicas de Minas Gerais, Belo Horizonte, Minas Gerais Brazil; ^2^ Rede Mater Dei de Saúde, Belo Horizonte, Minas Gerais Brazil

## Abstract

**Background:**

Frailty syndrome is a prevalent condition associated with high‐impact outcomes for the elderly, such as falls, hospitalization, functional loss and mortality. It can be triggered by several clinical conditions and dementia is one of them. More studies are needed to deeply understand the relationship between these two conditions. Frailty is prevalent in patients with advanced dementia, but this study was developed with the objective to understand if patients with mild cognitive impairment have increased risk for developing frailty too.

**Method:**

This is a cross‐sectional study in which 87 community‐dwelling elderly people were included and evaluated in relation to the presence of cognitive impairment and frailty. Patients with severe cognitive impairment, assessed by a score less than 6 in the CS‐10 (Point Cognitive Screening) were excluded. Individuals with a score between 6 and 7 on the CS‐ 10 were considered to have mild or moderate cognitive impairment and participants with a score greater than 7 on this test were considered to have no cognitive impairment. The individuals were evaluated for the presence of frailty based on the criteria proposed by Fried. Individuals with a score greater than or equal to 3 were considered frail, with score between 0 and 2 were considered non‐frail. In order to evaluate the correlation and association between the characteristics, Pearson’s chi‐square test was calculated. If the p‐value was lower than the significance level of 0.05, it is possible to conclude the association between the development of frailty and a worse score in CS‐10.

**Result:**

In this sample, 50.6% were frail. The mean score of the patients in CS 10 was 8.2. 69.2% of frail patients had a CS10 score between 6 and 7 points. At the highest score range above 10 points, we have that 72% are from non‐frail patients. Thus, there is an association between the risk of frailty and the CS10 score (p‐value 0.008). The Kruskal Wallis test, which evaluates the difference between the medians, also indicates statistically significant association between them (p‐value 0.002).

**Conclusion:**

Patients with mild cognitive impairment have an increased risk of developing frailty syndrome.